# The role of non‐pharmaceutical interventions on influenza circulation during the COVID‐19 pandemic in nine tropical Asian countries

**DOI:** 10.1111/irv.12953

**Published:** 2022-01-08

**Authors:** William W. Davis, Joshua A. Mott, Sonja J. Olsen

**Affiliations:** ^1^ Influenza Division Centers for Disease Control and Prevention Atlanta Georgia USA; ^2^ Thailand MOPH‐US CDC Collaboration Nonthaburi Thailand

**Keywords:** Asia, COVID‐19, non‐pharmaceutical interventions, NPIs, seasonal influenza

## Abstract

**Background:**

Low global influenza circulation was reported during the coronavirus‐19 pandemic. We explored relationships between non‐pharmaceutical interventions (NPIs) and influenza in tropical Asian countries.

**Methods:**

Using World Health Organization (WHO) surveillance data from 2015 to 2019 and the WHO shiny app, we constructed expected seasonal influenza epidemic curves from March 2020 to June 2021 and compared the timing, and average percent positivity with observed data. We used multivariate regression to test associations between ordinal NPI data (from the Oxford Stringency Index) 4 weeks before the expected 2020/21 epidemics and present adjusted incidence rate ratio (IRR) or relative proportion ratio (RPR) and 95% confidence intervals (CI).

**Results:**

Data from nine countries predicted 18 seasonal epidemics; seven were observed. Five started 6–24 weeks later, and all were 4–21 weeks shorter than expected. Five epidemics had lower maximum peak values (percent positivity), and all but one had lower average percent positivity than expected. All countries implemented NPIs. Each increased level of school closure reduced risk of an epidemic by 43% (IRR = 0.57, CI: 0.34, 0.95). Each increased level of canceling public events reduced the average percent positivity across the season by 44% (RPR = 0.56, CI: 0.39, 0.82) and each increased level in restricting internal movements reduced it by 41% (RPR = 0.59, CI: 0.36, 0.96). Other NPIs were not associated with changes.

**Conclusions:**

Among nine countries, the 2020/21 seasonal epidemics were delayed, shorter, and less intense than expected. Although layered NPIs were difficult to tease apart, school closings, canceling public events, and restricting internal movements before influenza circulation seemed to reduce transmission.

## 1 BACKGROUND

In 2020 and 2021, while cases of coronavirus‐19 (COVID‐19) surged, the world experienced historically low circulation of seasonal influenza viruses,^1^ likely due to non‐pharmaceutical interventions (NPIs) that were implemented to control COVID‐19.^2^ NPIs such as face masks, hand hygiene promotion, school and workplace closing, and other social distancing measures intended to disrupt transmission of severe acute respiratory syndrome coronavirus 2 (SARS‐CoV‐2) can also disrupt transmission of influenza.^3^

To track implementation of NPIs, the Oxford School of Government developed the Oxford Stringency Index (OSI),^4^ a composite measure of 23 interventions including social distancing, travel, and mask regulations measured at national and subnational levels. Within a country, the geographic coverage and degree of stringency are determined to generate a score for each NPI. Scores for individual NPIs are combined and normalized to a scale of 1 to 100 to create the OSI. OSI levels were positively associated with COVID‐19 deaths averted in Europe.^5^

We hypothesized that NPIs implemented for SARS‐CoV‐2 were associated with reduced influenza circulation in tropical Asian countries, knowing that in 2020 influenza circulation was reduced in the region, but that some countries still experienced seasonal epidemics. We hypothesized that countries that implemented more stringent mitigation policies during the COVID‐19 pandemic before the start of their “typical” or expected influenza season would have lower or delayed influenza circulation compared with those with lower stringency or later implementation.

## 2 METHODS

Based on prior work,^6^ we identified nine tropical countries in Asia that consistently (>50% of weeks) reported surveillance data from 2015 to 2021 into the WHO FluMart global repository for influenza epidemiology and laboratory surveillance data,^7^ as well as OSI data for 2020 and 2021. We extracted publicly available data from FluMart and OSI from January 2016 through June 2021. Data were downloaded on July 22, 2021 and included the following: the number of surveillance samples tested per week for influenza, number testing positive for influenza viruses, influenza virus type, subtypes and lineages, and the OSI and 10 of its components (school closures, workplace closures, canceling public events, restrictions on gatherings, closing public transportation, stay at home requirements, restrictions on internal movements; that is, inter‐city and inter‐provincial travel, international travel controls, public information campaigns, and mask mandates). We used percent positivity (samples testing positive/samples processed) by week as an indicator of intensity of influenza transmission.

We constructed typical seasonal influenza epidemic curves for each country using 5 years (2015–2019) of surveillance data using the WHO R shinyapp.^8^ Briefly, the app identifies and aligns seasonal curves so that all peaks occur on the date that is the mean of the peak dates from each year, and creates a typical seasonal epidemic curve that is the average percent positive for all aligned historical curves. The app generates thresholds for season start and end (when percent positivity is greater than the median percent positivity for the year). For comparison, we repeated the analysis using the moving epidemic method (MEM).^9^ We present results from the WHO method as it allows for multiple seasonal epidemics each year (a more common occurrence in tropical countries) and estimates different thresholds for each epidemic.

Using the threshold values from the typical seasonal epidemic curves, we identified the start week, end week, and peak intensity week for each epidemic in the typical season (for years 2015–2019) and the seasonal epidemics between March 2020 and June 2021. We determined presence or absence of a season if 2020/21 peaks crossed the epidemic threshold. We calculated delay in start and peak weeks for 2020/21 seasons and the change in season length in weeks, compared with typical seasons. We calculated peak intensity, defined as the highest percent positivity in the season and the average percent positivity across all weeks in each 2020/21 influenza season, or across the typical season time period if there was no 2020/21 season. We calculated the average proportion of respiratory specimens that were positive for influenza across the baseline 5 years, in 2020 after Week 10 and used Kruskal–Wallis tests to compare the means because the distribution of the proportion testing positive is often non‐parametric. We used *t* tests to compare average percent positivity between seasons and *z* tests to compare observed 2020/21 peak intensity (positive samples in the peak week/samples tested for the peak week) with the combined peak intensities for the 2015–2019 peaks (total positive samples for each 2015–2019 peak week/total samples tested for each 2015–2019 peak week).

For regression analyses, we used OSI or 10 component NPIs as independent variables and measures of influenza circulation (presence of 2020/21 peak, intensity of the 2020/21 peaks, and average percent positivity during the 2020/21 season or typical season time period) as dependent variables. We used influenza and OSI data from the 2020 and 2021 seasons, starting at Week 10 in 2020 (week starting March 1, 2020), the earliest date for when NPIs were first implemented for COVID‐19 in these countries. We used values for the OSI and its 10 component NPI variables 4 weeks before the typical influenza season and 4 weeks before the 2020 and 2021 seasons began. If there was no 2020 season, we used the OSI or NPI value 4 weeks before the typical season started. Published data suggests that COVID‐19 cases drop 3 weeks after implementing NPIs, and because influenza is less transmissible than COVID‐19, we determined that a 4‐week lag would be more than efficient to measure the impacts of NPIs on influenza.^10^

NPI variables were ordinal, with values ranging from 0–2 to 0–4 that represented the intensity of the intervention. Half steps (+0.5) indicated the geographic scope of the intervention (no half step = partial coverage and half step = full national coverage). For example, for school closings, ordinal values were as follows: 0, no measures; 1, recommend closing or all schools open with alterations resulting in significant differences compared with non‐COVID‐19 operations in limited geographic areas; 1.5, same as 1 but implemented nationally; 2, require closing (only some levels or categories, e.g., just high school or just public schools) in limited geographic areas; 2.5, same as 2 but implemented nationally; 3, require closing all levels in a limited geographic area; and 3.5, as 3 but implemented nationally. We transformed the ordinal scales by doubling them and subtracting one so that they contained only whole numbers. We generated a correlation matrix with the STATA *correlate* command to examine relationships between NPI variables.

We used generalized linear models to test associations between levels of OSI or NPIs 4 weeks prior to the start of the typical or 2020/21 influenza seasons and characteristics of the 2020/21 influenza seasons, including presence of 2020/21 peak (to indicate if a season occurred or not), intensity of the 2020/21 peaks, and average percent positivity during the 2020/21 season or typical season time period (to indicate a blunting of the intensity of the influenza season). All models controlled for population density. For presence of 2020/21 peaks as the dependent variable, we used a generalized linear model with Poisson family, log link, and robust variance estimates to estimate the adjusted incidence rate ratio (IRR, or relative risk) and 95% confidence intervals (95% CIs) of having a 2020/21 season. Values for percent positivity across the 2020/21 seasons and maximum peak value fell between 0 and 1; thus, regressions with these variables as the dependent variables used generalized linear models with logit link, binomial family, and robust option in STATA for error estimates. The exponentiated coefficients of the regression terms give the relative proportion ratio (RPR), or the ratio of the proportion of surveillance samples testing positive for influenza with an NPI intervention to the proportion of samples testing positive at baseline. Analyses were done with STATA 16, the WHO shiny app, and the MEM package in R.

## 3 RESULTS

**FIGURE 1:**
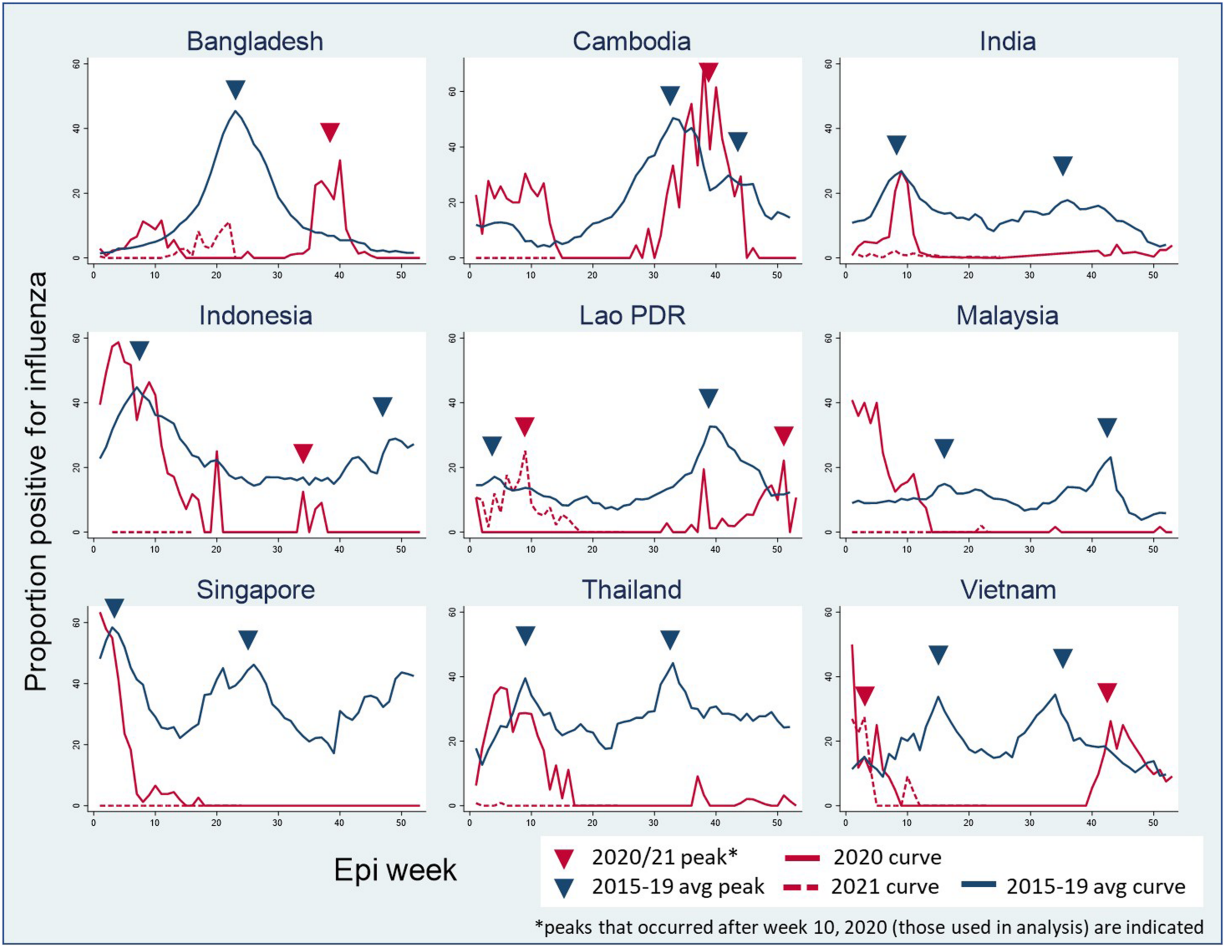
Seasonal (2020–2021) and average (2015–2019) influenza curves and peaks by epi week

Nine countries had OSI data for 2020 and 2021 and had reported >50% of weeks surveillance data from 2015 to 2021 into the WHO FluMart repository.^7^ Between Week 10, 2020 and Week 25, 2021, one country (Vietnam) had three seasonal epidemics, seven countries (Indonesia, India, Cambodia, Lao People's Democratic Republic [PDR], Malaysia, Singapore, Thailand, and Vietnam) had two typical seasonal influenza epidemics, and one (Bangladesh) had one seasonal epidemic (Table 1, Figure S1). Of 18 expected influenza epidemics in these nine countries, 11 epidemics (61%) in seven countries (Indonesia, India, Cambodia, Malaysia, Singapore, Thailand, and Vietnam) did not occur in 2020 or 2021 (Figure 1). Of the seven epidemics (in Bangladesh, Indonesia, Cambodia, Lao PDR, and Vietnam) that did occur, five had delayed starts ranging from 6 to 24 weeks from the typical season, and two (Indonesia and Vietnam) started before the typical epidemic start. All seven epidemics were 4–21 weeks shorter than the typical epidemic. Five of seven seasons had peak intensity lower than the typical epidemic (51% to 88% of the average peak, *p* values 0.04 to 0.18), and one seasons had a peak about the same as the typical epidemic (104% in Cambodia, *p* = 0.65), and one season had a peak slightly greater than the typical season (104% Lao PDR, *p* = 0.65, Table 1).

**TABLE 1:** Parameters of typical and 2020/21 influenza seasons, by country

		Start week		Peak week		End week		Season length (weeks)		Average percent positivity across season			Peak intensity (% positive)		
Country	Year	Typical season	During pandemic	Typical season	During pandemic	Typical season	During pandemic	Typical season	During pandemic	Typical season	During pandemic	*p*	Typical season	During pandemic	*p*
Bangladesh	2020	11	35	23	38	39	42	28	7	20.3	16.5	0.7	45.4	23.1	<0.01
Indonesia	2020	41	35	43	35	45	35	4	0	32.9	0	0.08	23.3	20.5	0.18
Indonesia	2021	1		7		17		16	0	21.1	0	<0.001	44.8	0	
India	2020	29		36		44		15	0	16.1	2	0.002	17.9	0.0	
India	2021	4		9		18		14	0	14.8	0.8	<0.001	26.8	0.0	
Cambodia	2020	20	31	30	38	40	44	20	13	31.5	36.9	0.44	54.8	56.9	0.65
Cambodia	2020	40		42		47		7	0	23.9	24.1	0.96	29.8	0.0	
Lao PDR	2020	31	48	39	50	49	51	18	3	15.1	14.9	0.78	32.6	15.5	0.02
Lao PDR	2021	51	5	4	8	11	11	12	6	19.4	13.1	0.1	17.3	17.9	0.04
Malaysia	2020	33		43		45		12	0	8.2	0.1	<0.001	23.2	0.0	
Malaysia	2021	6		16		27		21	0	10.2	0.1	<0.001	14.9	0.6	
Singapore	2020	20		27		31		11	0	41.2	0	<0.001	55.3	0.0	
Singapore	2021	50		5		12		14	0	45	0	<0.001	58.1	0.0	
Thailand	2020	25		33		50		25	0	28.3	0.7	<0.001	44.2	0.0	
Thailand	2021	6		9		14		8	0	29.6	0	<0.001	39.5	0.0	
Vietnam	2020	12		15		27		15	0	28.3	0	<0.001	37.5	0.0	
Vietnam	2020	28	42	34	44	42	47	14	5	25.4	20.7	0.96	34.5	23.0	0.04
Vietnam	2021	12	1	15	2	27	4	15	3	28.3	22.1	0.003	37.5	25	0.03

Abbreviation: Lao PDR, Lao People's Democratic Republic.

The average number of respiratory surveillance specimens tested per year from 2015 to 2019 ranged from 2437 in Vietnam to 20,990 in India; in 2020 all countries except Singapore tested 4% to 61% fewer specimens for influenza compared with the previous 5 years' average (Table 2). The average proportions of surveillance respiratory specimens testing positive for influenza across Weeks 10–52 in 2020 were lower in all countries compared with their previous 5 years' averages (*p* < 0.01 for all countries, Table 2). Similarly, the circulation of influenza outside of the seasons (determined by the WHO method) was also lower than the previous 5 years' averages (Figure 1).

**TABLE 2:** Average proportion of surveillance specimens positive for influenza, by year

	Specimens tested			Proportion positive for influenza		
Country	2015–2019 average	2020	Change (%)	2015–2019 (Weeks 10–53)	2020 (Weeks 10–53)	*p*
Bangladesh	7104	5534	−22.1	14.6	3.9	<0.001
Indonesia	3556	1376	−61.3	22.2	4.5	<0.001
India	20,990	10,715	−49.0	12	3.2	<0.001
Cambodia	1332	1136	−14.7	23.3	14.2	0.002
Lao PDR	4522	2505	−44.6	14.3	3.2	<0.001
Malaysia	4380	3161	−27.8	9.9	1.2	<0.001
Singapore	2628	3234	23.1	35.3	0.5	<0.001
Thailand	3113	2981	−4.2	26.1	2.8	<0.001
Vietnam	2437	2029	−16.7	22.1	4.6	<0.001

Abbreviation: Lao PDR, Lao People's Democratic Republic.

OSI composite scores increased sharply in all countries around Week 10 of 2020. Indonesia, Singapore, and Vietnam had initiated NPIs before Week 10, as indicated by the OSI score. The level of implementation of NPIs dropped between Weeks 20 to 30 in 2020 in most countries, and fluctuated after that, presumably in response to waves of COVID‐19. Levels of individual NPIs varied by the strictness with which they were applied, timing, and country.

Influenza A(H3N2) and A(H1N1)pdm09 viruses accounted for 89% to 100% of viruses identified in countries with influenza epidemics (i.e., Bangladesh, Indonesia, Cambodia, Lao PDR, and Vietnam) from Weeks 10 to 52, 2020 (Table 3). During the same period, influenza A(H1N1)pdm09 viruses were detected in all nine countries, although at a lower percentage than A(H3N2), except in India and Thailand. Influenza B (Victoria) viruses were detected in Cambodia and Thailand, and no influenza B (Yamagata) viruses were detected in any country. In the first 6 months of 2021, influenza A(H3N2) and B (Victoria) were the predominant viruses, accounting for 99% to 100% of viruses identified in countries with influenza epidemics that year. Only India and Lao PDR reported any A(H1N1)pdm09. No influenza B (Yamagata) viruses were detected.

**TABLE 3:** Influenza viruses circulating in 2020 and 2021

		Circulating influenza viruses just before COVID‐19 pandemic (Week 1 2019 to Week 10 2020)					Circulating influenza viruses during the COVID‐19 pandemic (Weeks 10–52, 2020)					Circulating influenza viruses during the COVID‐19 pandemic (Weeks 1–26, 2021)				
Country	Influenza peak during COVID 19 pandemic	% A (H1pdm09)	% A(H3)	% B Yam	% B Vic	Total number of positive samples	% A (H1pdm09)	% A (H3)	% B Yam	% B Vic	Total number of positive samples	% A (H1pdm09)	% A (H3)	% B Yam	% B Vic	Total number of positive samples
Bangladesh	Y	22.1	33.4	4.2	39.7	1866	12.2	87.8	0.0	0.0	245.0	0.0	1.2	0.0	98.8	83.0
Indonesia	Y	48.5	23.8	2.7	24.9	811	1.6	98.4	0.0	0.0	128.0	0.0	0.0	0.0	0.0	0.0
India	N	60.7	27.9	1.0	5.0	11,004	71.9	17.2	0.0	0.5	192.0	8.2	89.8	0.0	1.5	196.0
Cambodia	Y	46.6	27.0	0.3	2.3	365	31.5	57.6	0.0	10.9	92.0	0.0	0.0	0.0	100.0	1.0
Lao PDR	Y	27.5	25.8	1.0	27.5	596	0.8	99.2	0.0	0.0	123.0	0.8	99.2	0.0	0.0	123.0
Malaysia	N	34.7	14.1	0.0	0.4	1376	33.3	51.1	0.0	0.0	45.0	0.0	100.0	0.0	0.0	1.0
Singapore	N	51.2	18.9	1.9	25.6	1513	33.3	4.8	0.0	0.0	21.0	0.0	0.0	0.0	0.0	0.0
Thailand	N	28.8	24.4	0.7	42.9	1804	61.4	30.7	0.0	3.4	88.0	0.0	100.0	0.0	0.0	2.0
Vietnam	Y	53.2	4.5	2.6	14.6	378	0.0	100.0	0.0	0.0	123.0	0.0	100.0	0.0	0.0	39.0

Abbreviation: Lao PDR, Lao People's Democratic Republic.

Using multivariate analysis and controlling for population density, we found that school closings were significantly associated with absence of a seasonal influenza epidemic (Table 4). For each step increase in school closings 4 weeks before the expected influenza season, the risk of having an influenza season between January 2020 and June 2021 dropped by 43% (IRR: 0.57, 95% CI: 0.34–0.95, *p* = 0.03). Overall OSI as a composite measure for NPIs was a borderline associated with a decreased risk of having an influenza season. For each one‐point increase in OSI, the risk of having an influenza season decreased by 2% (IRR: 0.98, 95% CI: 0.96–1, *p* = 0.02). The population density term was significant (*p* < 0.05) seven of 11 regression models, including those for OSI and school closings.

**TABLE 4:** Multivariate associations between non‐pharmaceutical measures and influenza transmission

Multivariate associations between non‐pharmaceutical interventions* and presence (yes/no) of a seasonal influenza epidemic between January 2020 and June 2021, controlling for population density					Multivariate associations between non‐pharmaceutical interventions* and percent of respiratory specimens positive for influenza across 2020/21 influenza epidemics,** controlling for population density				
Model#	Variable	IRR	*p*	95% CI	Model#	Variable	RPR	*p*	95% CI
1	Oxford Stringency Index (OSI)**	0.98	0.02	0.96, 1	12	Oxford Stringency Index (OSI)***	0.97	0.1	0.93, 1.01
2	**School closings**	**0.57**	**0.03**	**0.34, 0.95**	13	School closings	0.94	0.71	0.69, 1.29
3	Workplace closures	0.86	0.29	0.64, 1.14	14	Workplace closures	1.51	0.1	0.92, 2.48
4	Canceling public events	0.81	0.27	0.56, 1.17	15	**Canceling public events**	**0.56**	**>0.01**	**0.39, 0.82**
5	Restrictions on gatherings	0.91	0.32	0.76, 1.09	16	Restrictions on gatherings	0.93	0.37	0.79, 1.09
6	Closing public transport	0.69	0.29	0.34, 1.38	17	Closing public transport	0.77	0.47	0.38, 1.57
7	Stay at home orders	0.69	0.13	0.42, 1.12	18	Stay at home orders	0.90	0.67	0.55, 1.47
8	Restrictions on internal movements	0.89	0.55	0.61, 1.3	19	**Restrictions on internal movements**	**0.59**	**0.04**	**0.36, 0.96**
9	International travel restrictions	0.73	0.47	0.32, 1.71	20	International travel restrictions	0.41	0.1	0.14, 1.19
10	Public information campaigns	1.00	NA	NA	21	Public information campaigns	0.00	NA	NA
11	Mask mandates	1.00	0.98	0.79, 1.27	22	Mask mandates	0.88	0.34	0.67, 1.15
*measured 4 weeks prior to the typical start of the influenza epidemic**OSI is a composite measure of 23 individual COVID‐19 non‐pharmaceutical interventions					*measured 4 weeks prior to the typical start of the influenza epidemic** for countries with no epidemic, dates for the typical epidemic season were used***OSI is a composite measure of 23 individual COVID‐19 non‐pharmaceutical interventions				

Abbreviations: CI, confidence interval; OSI, Oxford Stringency Index; RPR, relative proportion ratio.

*Note*: Bold indicates variables that had significant results in the multivariate analysis.

In multivariate analyses, also controlling for population density, canceling public events and restrictions on internal movements were associated with reductions in percent positivity during the 2020/21 epidemics or the absence of an epidemic during the weeks in 2020/21 when the typical seasonal epidemic was expected. For each step increase in canceling public events (i.e., no measures, recommend canceling locally/nationally, and require canceling locally/nationally), the average percent positivity across the influenza season decreased by 44% (RPR: 0.56, 95% CI: 0.39–0.82, *p* = >0.01) compared with baseline (no NPI intervention). Similarly, for each step increase in restrictions on movements (no measures, recommend not to travel domestically locally/nationally; restrictions in place locally/nationally), the average percent positivity across the influenza season decreased by 41% (RPR: 0.59, 95% CI: 0.36–0.96, *p* = 0.04) compared with no intervention. No other NPI was associated with a change in influenza activity. The population density term was significant (*p* < 0.05) in six of 11 regression models, including the model for restrictions on internal movements. A correlation matrix of NPIs indicated that no two variables were highly correlated (correlation ≥ ± 0.8), and the variables with the highest correlation were canceling public events and restrictions on internal movements (correlation coefficient = 0.67) (Table S1).

## 4 DISCUSSION

The countries in our analysis experienced fewer epidemics and less intense influenza transmission in 2020 and 2021 compared with the five previous years. Only Cambodia and Lao PDR each had one seasonal peak with similar intensity to the typical seasonal peak. Influenza A(H3N2) was the predominant virus circulating in countries that had seasonal peaks in 2020 and the first half of 2021. School closures 4 weeks prior to the expected influenza season were statistically significantly associated with a decreased risk of having an influenza season, controlling for population density. Canceling public events and restrictions on internal movements were associated with decreases in average percent positivity during 2020/21; that is, blunting the intensity of the season, after controlling for population density.

Many ecological studies have documented the effects of broad mitigation strategies on influenza circulation.^2^ However, given the concurrent and overlapping application of mitigation measures, it has been hard to tease out the role or contribution of each NPI to decreased influenza circulation. As others have speculated, cessation of global travel likely eliminated reseeding events,^11^ and other measures likely prevented influenza from propagating within countries. By using the OSI and its separate components, we were able to show that school closures decreased the risk of having a seasonal epidemic and canceling public events and restricting movements decreased the magnitude of that epidemic. These findings are consistent with what we know about influenza transmission.

Several studies previously documented disruption of seasonal and pandemic influenza during school holidays and other school closures.^12^ One proposed mechanism is increased and prolonged influenza virus shedding in children compared with adults' results in high transmissibility between children at schools who then infect their family members.^13^

Influenza outbreaks have been documented at public events such as mass gatherings.^14^ However, data on the effects of restricting mass gatherings on the impact of influenza transmission are limited.^3^ A modeling study suggests that restricting mass gatherings just prior to the seasonal peak may reduce influenza transmission, but the impacts may be mild.^15^ A review suggests that mass gatherings may facilitate seeding viruses into new populations during a pandemic and that restricting mass gatherings in combination with other NPIs may contribute to reducing transmission.^16^ In our analysis, we found that restrictions on mass gatherings were associated with reduced influenza circulation. It is possible that other NPIs are also necessary for these restrictions to have an impact, but we were not able to explore this further.

Modeling studies indicate that spread of pandemic influenza in the United States in 2009 occurred primarily through local transmission,^17^ and models of seasonal influenza in Australia also suggest local diffusion of influenza is a driver of seasonal epidemics, combined with importation of cases from abroad.^18^ One modeling study suggests that restrictions on movement are only effective in reducing seasonal influenza transmission if at least 50% of movements are restricted.^19^

We did not find associations between mask mandates and reduction in influenza circulation. There are no population‐level mask use studies for direct comparison; however, in systematic review, 10 randomized controlled trials outside of the healthcare setting examining the effect of mask use on influenza infections when worn by ill persons or when worn by well persons to prevent infection showed no effect.^20^ In another meta‐analysis that included observational studies, there was some evidence suggesting mask use was protective against influenza virus infection in some settings.^21^ Several studies in household and community settings suggest that in these settings, adherence to mask wearing is variable,^22^ and that other interventions, such as handwashing, may be needed in conjunction with masks to reduce influenza transmission.^23^ We also found no association between reduced influenza activity and border closures. However, it is possible that early, extensive border closures might have prevented influenza viruses from being reintroduced, but that our analysis would not uncover this association unless the timing occurred 1 month before the expected influenza epidemic.

Circulation of predominately influenza A(H3N2) viruses in Southeast Asia during the COVID‐19 pandemic has been previously reported.^6^ Influenza A(H3N2) viruses are thought to evolve in this region, and it is possible that endemic circulation of these viruses contributed to seasonal outbreaks.^24^ This finding contrasts with the rest of the world, where low level circulation was predominately influenza B viruses.^7^

Cambodia, Laos PDR, and Vietnam all reported seasonal influenza peaks, despite having implemented NPIs. These countries detected very few or no COVID‐19 cases until later in the pandemic (March to June 2021). Similarly, Singapore was able to reduce COVID‐19 cases in September 2020, after experiencing an initial wave of transmission. This suggests that either these countries' border closures were successful in preventing introduction of SARS‐CoV‐2 or that NPIs implemented inside the countries were successful in stopping transmission, or a combination of both. If these interventions were able to control SARS‐CoV‐2, they should have also controlled influenza during the same time periods. This was the case in Singapore and Thailand, but not in Cambodia, Lao PDR, or Vietnam. Cambodia and Lao PDR, after a period reduction in influenza circulation, experienced influenza peaks similar to the typical seasonal peaks, although both countries had lower influenza circulation in 2020–2021 compared with previous years. Vietnam also experienced seasonal peaks, although reduced in intensity compared with the typical seasonal peaks. Variable adherence to NPIs and inaccuracy in measuring NPIs might explain these findings, although data are limited on this; one study in Vietnam reported high compliance with NPIs.^25^

This study has several limitations. Our sample size of 18 typical seasons in 9 countries was limited; a larger dataset would enable examination beyond the multivariate regressions that we used. Bans on international travel, in particular, may modify the effects of NPIs on influenza if they prevent seasonal influenza from ever entering a country. We were not able to explore this further, although we found no association with border closure and influenza circulation. We also did not explore temperature and humidity variations, which are known to affect transmission. We did not explore variation in income status of countries, which may have affected implementation and adherence to control policies. Influenza surveillance was affected by the COVID‐19 pandemic as resources were shifted to outbreak response, resulting in fewer respiratory samples tested for influenza in all countries except Singapore, compared with previous years. Health‐seeking behaviors also changed during the pandemic and could have decreased consultation or influenza detection rates; several reports suggest that people were less likely to seek care at clinics because of fears of contracting COVID‐19.^26^ Our analysis was limited to positive tests for influenza only; data were not available for subanalyses of other demographic factors of positive cases (e.g., age). Influenza is identifiable year‐round and may have less well‐defined peaks compared with seasonality in temperate climates. Although we used standard methodology to define seasonality, and we allowed for two peaks per season, it is possible that our approach of examining discrete seasons does not exactly reflect the reality of influenza circulation in tropical climates. Our model examined relationships between levels of NPIs implemented 4 weeks before expected or observed influenza seasons, thus capturing only a cross‐sectional perspective of the relationship between influenza circulation and NPIs. It is likely that a more complex relationship between NPIs and influenza circulation exists. We did not account for adherence to OSI measures, and there were likely discrepancies in how they were implemented between and within countries. However, if there was low adherence, we would have expected more influenza circulation. Finally, there was some heterogeneity in the pharmaceutical interventions in place, most notable influenza vaccination coverage levels; however, overall coverage in the region is low (~ <7% of total population in each country).^27^

Our analysis found that after controlling for population density, closing schools 4 weeks before an expected influenza season was associated with reduced influenza circulation. Similarly, after controlling for population density at the country level, we found that canceling public events and placing restrictions on internal movements decreased the intensity of influenza circulation. At the population level, we found no association between mask use and influenza circulation; however, further exploration is warranted as mask mandates may be easier to implement than other NPIs. These findings may help inform policies to control future seasonal and pandemic influenza epidemics. As the COVID‐19 pandemic wanes and NPIs are lifted, it will be important to continue to collect data on NPIs to learn as much as we can about their effect on influenza circulation.

## AUTHOR CONTRIBUTIONS

**William Davis:** Data curation; formal analysis; investigation; methodology; software; validation; visualization. **Joshua Mott:** Investigation; methodology. **Sonja Olsen:** Conceptualization; investigation; methodology; supervision.

## DISCLAIMER

The findings and conclusions in this report are those of the author(s) and do not necessarily represent the official position of the Centers for Disease Control and Prevention (CDC).

### PEER REVIEW

The peer review history for this article is available at https://publons.com/publon/10.1111/irv.12953.

## Supporting information


**Figure S1.** Proportion positive for influenza (2015‐2019) and average seasonal curves by epi week

**Table S1.** Correlation matrix of OSI component variables

## Data Availability

All data used in this study are publicly available at these urls: https://apps.who.int/flumart/Default?ReportNo=12 https://www.bsg.ox.ac.uk/research/research-projects/covid-19-government-response-tracker#data.
