# Supplementary figures and images for: The role of non‐pharmaceutical interventions on influenza circulation during the COVID‐19 pandemic in nine tropical Asian countries
Source: Influenza Other Respir Viruses. 2022 Jan 8;16(3):568–76. doi: 10.1111/irv.12953 (PMC8983905; doi:10.1111/irv.12953)

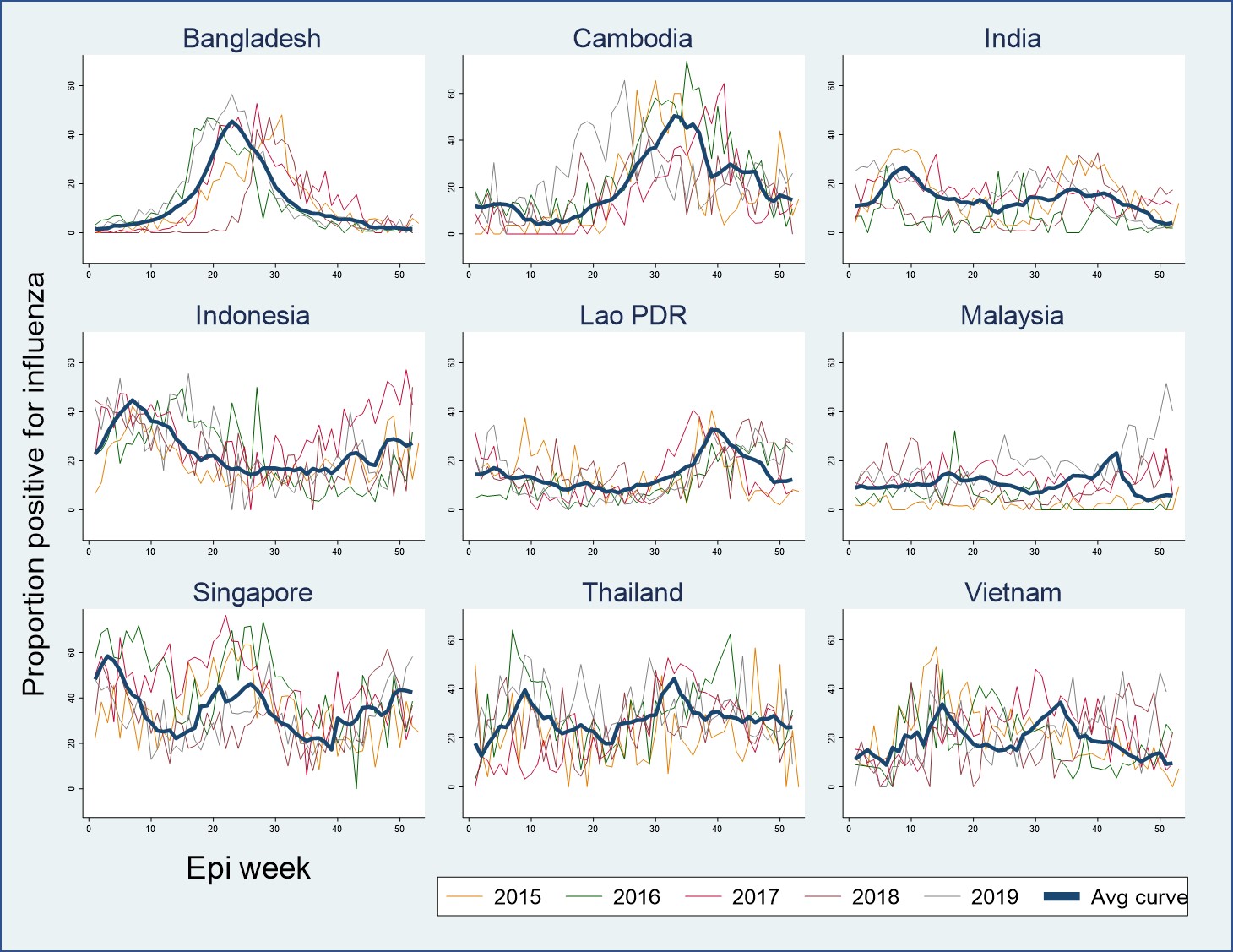

Supplement: Supplementary file 1 — Figure S1. Proportion positive for influenza (2015‐2019) and average seasonal curves by epi week [file IRV-16-568-s001.jpg]
