# Supplementary material for: The role of non‐pharmaceutical interventions on influenza circulation during the COVID‐19 pandemic in nine tropical Asian countries
Source: Influenza Other Respir Viruses. 2022 Jan 8;16(3):568–76. doi: 10.1111/irv.12953 (PMC8983905; doi:10.1111/irv.12953)
Supplement: Supplementary file 2 — Table S1. Correlation matrix of OSI component variables [file IRV-16-568-s002.docx]

**Table S1**

| **Table S1. Correlation matrix of OSI component variables** | | |  |  |  |  |  |  |  |  |
| --- | --- | --- | --- | --- | --- | --- | --- | --- | --- | --- |
|  | School closings | Workplace closures | Cancelling public events | Restrictions on gatherings | Closing public transport | Stay at home orders | Restrictions on internal movements | International travel restrictions | Public information campaigns | Mask mandates |
| School closings | 1.00 |  |  |  |  |  |  |  |  |  |
| Workplace closures | 0.31 | 1.00 |  |  |  |  |  |  |  |  |
| Cancelling public events | 0.48 | 0.35 | 1.00 |  |  |  |  |  |  |  |
| Restrictions on gatherings | 0.38 | 0.35 | 0.61 | 1.00 |  |  |  |  |  |  |
| Closing public transport | 0.19 | 0.01 | 0.28 | 0.55 | 1.00 |  |  |  |  |  |
| Stay at home orders | 0.43 | 0.48 | 0.14 | 0.40 | 0.30 | 1.00 |  |  |  |  |
| Restrictions on internal movements | 0.43 | 0.27 | 0.67 | 0.59 | 0.43 | 0.21 | 1.00 |  |  |  |
| International travel restrictions | 0.15 | 0.24 | 0.62 | 0.51 | 0.40 | 0.31 | 0.49 | 1.00 |  |  |
| Public information campaigns | . | . | . | . | . | . | . | . | . |  |
| Mask mandates | -0.17 | 0.24 | 0.11 | 0.24 | 0.18 | 0.41 | 0.01 | 0.29 | . | 1.00 |
